# Cytokines in HIV-1 patients on combination antiretroviral therapy with persistent low-level viremia at a tertiary hospital in Western Kenya

**DOI:** 10.11604/pamj.2025.51.18.47628

**Published:** 2025-05-21

**Authors:** Rose Undisa, Isaac Ndede, Lameck Oteko Diero

**Affiliations:** 1 Department of Pathology, Moi University, Eldoret, Kenya,; 2 Department of Medicine, Moi University, Eldoret, Kenya

**Keywords:** Persistent low-level viremia, HIV-1, combination antiretroviral therapy, cytokines

## Abstract

**Introduction:**

persistent low-level viremia (pLLV) in HIV-1 participants on combination antiretroviral therapy (cART) is a significant predictor of immune activation and virologic rebound. Elevated cytokine levels are linked to pLLV, yet the roles of pro- and anti-inflammatory cytokines remain unclear in settings such as Kenya. This study assessed IL-17, IFN-γ, IL-10, and TGF-β in HIV-1 pLLV and virally suppressed participants at Moi Teaching and Referral Hospital (MTRH) to explore alternative biomarkers for virologic monitoring in resource-limited settings.

**Methods:**

cross-sectional study of 82 age- and gender-matched HIV-1 participants on first-line cART, -41 pLLV (50-500 copies/ml), and 41 virally suppressed (<50 copies/ml) was conducted using MTRH database data. Plasma cytokines (IL-17, IFN-γ, IL-10, TGF-β) were measured by ELISA (Zeptometrix, USA), and viral loads by RT-PCR (Abbott, USA). Data were analyzed using Mann-Whitney U, Chi-squared, and logistic regression tests.

**Results:**

the median (IQR) levels of IL-17, IFN-γ, IL-10, and TGF-β (pg/ml) were significantly higher in pLLV participants on first-line cART compared to virally suppressed participants: 23.8 (21.3-25.8) vs. 15.5 (12.7-18.2); 28.3 (23.5-31.2) vs. 11.4 (8.8-14.8); 45.2 (34.0-53.7) vs. 28.4 (22.6-31.6); and 56.9 (50.0-67.8) vs. 27.7 (19.5-34.7) p<.001. The odds (95% CI) of being suppressed were reduced by TGF-β 0.329 (0.035-0.361), IFN-γ, 0.360 (0.270-1.501, and IL-17 0.938 (0.691-1.273), but increased by IL-10 1.106 (0.675-1.811), though not statistically significant.

**Conclusion:**

persistent low-level viremia (pLLV) presents with elevated pro-inflammatory (IL-17, IFN-γ) and anti-inflammatory cytokines (IL-10, TGF-β) levels. Recommendation: exploring other cytokines in pLLV could enhance understanding of cytokine levels and viral suppression and may help refine HIV-1 treatment.

## Introduction

HIV-1 remains a significant global health challenge, with about 39 million people affected as of 2023 [1]. Eastern and Southeast Africa (ESA) regions bear the greatest burden, accounting for 20.8 million cases and the highest AIDS-related deaths at 260,000. Kenya, a part of ESA, had 1.4 million people living with HIV-1 and 18,000 AIDS-related deaths during the same period [1]. About 94% of Kenyan HIV-1 participants are on combination antiretroviral therapy (cART), and of these, 1.2 million reportedly achieve viral suppression, as required by Sustainable Development Goal 3.3. However, over 0.2 million do not achieve the desired suppression [1]. The primary goal of cART is to achieve undetectable viral loads and sustained viral suppression, leading to improved immune function and reduced transmission risk, thus decreasing HIV/AIDS-related morbidity and mortality [2]. Some HIV-1 participants on cART experience persistent low-level viremia (pLLV), characterized by a viral rebound between 50 and 500 copies/ml due to enduring virus reservoirs [3-5]. Persistent low-level viremia has been linked to virologic failure, often characterized by viral loads of up to >1,000 copies/ml after previously achieving suppression, which then increases the incidences of morbidity and mortality due to HIV-1 infection [6]. Participants with pLLV need to be identified as they require additional management strategies targeted to their specific needs, including drug regimen adjustments, adherence counseling, and close monitoring [7]. In Kenya and in the regions where HIV-1 is a significant public health challenge, effective monitoring of HIV-1 participants on cART for pLLV occurrence is crucial for optimizing care.

Access to viral load monitoring and testing remains limited due to cost in resource-constrained settings, particularly in public hospitals and facilities in rural areas [8,9]. These challenges underscore the need for alternative monitoring methods, such as assessing immune biomarkers indicative of inflammation, immune activation, and possible virologic failure. Monitoring cytokine profiles can offer valuable insights into inflammation, immune activation, and, therefore, viral replication and rebound in regions where viral load testing is less accessible [10,11]. The host immune system controls HIV-1 replication through interactions between pattern recognition receptors (PRRs) and pathogen-associated molecular patterns (PAMPs), triggering cytokine responses that control the virus through a balance of inflammation, immune activation, and anti-inflammatory responses [12,13]. The pro-inflammatory and anti-inflammatory cytokine balance is crucial for HIV-1 infection control and preventing tissue damage. This study examined pro-inflammatory (IL-17, IFN-γ) and anti-inflammatory (IL-10, TGF-β) cytokines in HIV-1 participants on first-line cART with pLLV and in suppressed participants on cART to find out if there is any relationship between the expression of these cytokines and pLLV status. This can then serve as a potential biomarker for viral rebound in pLLV. Specific objectives: to determine the IL-17, IFN-γ, IL-10, and TGF-β levels, and viral load in HIV-1 patients on first-line cART with pLLV and HIV-1-suppressed patients at MTRH.

## Methods

**Study site:** the study was conducted at the Academic Model Providing Access to Healthcare (AMPATH) adult HIV-1 clinic modules in Moi Teaching and Referral Hospital, Eldoret.

**Study design:** we conducted a cross-sectional, comparative study.

**Study setting, location, and relevant dates:** this study was conducted at Moi Teaching and Referral Hospital (MTRH), Eldoret, Kenya, a major HIV-1 care center under the AMPATH program. A retrospective review identified HIV-1 participants with post-suppression pLLV and HIV-1-suppressed participants from adult HIV-1 clinic case records. Through purposive sampling, participants who met the study criteria were selected. All eligible participants accessible via consecutive sampling were likewise incorporated. Potential participants underwent screening based on diagnosis and relevant time frames. Data collection, including venous blood sampling and clinical data extraction, was conducted from January 2021 to December 2022. Plasma cytokines (IL-17, IFN-γ, IL-10, TGF-β) were measured by ELISA (Zeptometrix, USA), and viral loads were assessed by RT-Polymerase chain reaction (PCR) (Abbott m2000 system, USA).

**Study participants:** HIV-1-infected adults aged 18-57 years on first-line cART and receiving routine care at the AMPATH clinic, Moi Teaching and Referral Hospital (MTRH), between January 2021 and December 2022, were retrospectively selected from the MTRH HIV database. Inclusion required a documented history of either persistent low-level viremia (≥2 consecutive viral loads of 50-500 copies/mL without subsequent suppression) or sustained viral suppression (<50 copies/mL) over 24 months. Exclusion criteria included unstable viremia, comorbidities (e.g., diabetes, cardiovascular disease), and self-reported autoimmune, immunodeficiency, or hypersensitivity disorders to control for potential immune-related confounding.

### Variables

**Outcome and exposure:** the primary outcome was virologic status, defined as viral suppression (<50 copies/mL) or pLLV (two consecutive HIV-1 RNA measurements of 50-500 copies/mL over 24 months), quantified by RT-PCR on the Abbott m2000sp/rt platform (Abbott Molecular, USA). The main exposure was pLLV, and the predictor variables were plasma concentrations of pro-inflammatory cytokines (IL-17, IFN-γ) and anti-inflammatory cytokines (IL-10, TGF-β), measured from venous plasma using Zeptometrix ELISA kits per standardized protocols.

**Confounding, effect of modification, and diagnostic criteria:** key confounders include CD4+ T cell count, duration on cART, treatment adherence, co-infections (e.g., TB, hepatitis), baseline viral load, nutritional status, and residual immune activation. Effect modification was not formally tested; however, duration on cART, immune reconstitution (CD4 recovery), and HIV-1 subtype or resistance mutations may have influenced cytokine effects on viral suppression. Diagnostic criteria were clearly defined: pLLV was a viral load of 50-500 copies/mL; viral suppression was <50 copies/mL. HIV-1 diagnosis was confirmed per standard clinical protocols.

**Data sources:** data were extracted from the MTRH HIV-1 treatment database. Viral load testing and cytokine assays were conducted in the same diagnostic lab using consistent reagents, equipment, and personnel. All participants had ≥2 viral load results confirming their classification.

### Laboratory methods

**ELISA for cytokines:** cytokines were analyzed by ELISA (Zeptometrix, Buffalo, NY, USA), following the manufacturer´s instructions. Each sample was analyzed in duplicate to ensure the accuracy and reliability of the results. ABC solution and TMB agent were pre-warmed at 37°C for 30 minutes. Samples and standards (0.1 ml) were added and incubated at 37°C for 90 minutes. Biotinylated antibodies were added and incubated for 60 minutes. After three TBS washes, the ABC solution was added and incubated for 30 minutes. Following five TBS washes, the TMB agent was added and incubated at 37°C in the dark for 20-30 minutes. The TMB stop solution was added, and O.D. at 450 nm was measured within 30 minutes.

**Polymerase chain reaction for viral load:** viral load was analyzed by polymerase chain reaction- Abbott Real-Time PCR HIV-1 RNA assay, version 4.0. A master mix with primers, nucleotides, buffer, and DNA polymerase was prepared. Template RNA was added, and thermal cycling was performed. PCR products were analyzed for target bands. Results were reported with amplification details.

**Bias control:** participants were matched on age and gender to control for confounding. Data on cART duration and comorbidities were abstracted but not adjusted for statistically. Standardized phlebotomy, processing, and blinded laboratory analysis were used to minimize selection, information, and measurement bias. All assays were run under uniform quality-controlled conditions.

**Sample size determination:** based on Pagano and Gauvreau, 2000 [14].


$$n=\frac{2\left(z\alpha +z\beta \right)2\delta^{2}}{d^{2}}$$

Where: n is the sample size required in each group; Zα is the desired level of statistical significance, 1.96 for a 95% CI; Zβ is the desired power of 0.84.δ is the standard deviation of the outcome variable; d is the difference in the means between cases and controls n=41 per group (41 cases and 41 for control). We recruited 82 HIV-1 patients on first-line cART, comprising 41 pLLV patients with 50-500 copies/ml and 41 age- and gender 1:1 matched, virally suppressed controls with <50 copies/ml.

**Quantitative variables handling:** quantitative variables, including plasma cytokine concentrations (IL-17, IFN-γ, IL-10, TGF-β) and HIV-1 viral load, were treated as continuous data. Due to non-normal distribution, cytokine levels were summarized using medians and interquartile ranges and compared between groups using the Mann-Whitney U test. Viral load was dichotomized based on clinical thresholds into pLLV (50-500 copies/mL) and virally suppressed (<50 copies/mL) groups to enable biologically relevant comparisons. Associations between cytokine levels and virologic status were assessed using logistic regression, with odds ratios estimated per unit increase in cytokine concentration.

**Statistical methods:** statistical analyses were conducted using non-parametric Mann-Whitney U tests to compare cytokine concentrations between the pLLV and virally suppressed groups, given the skewed distribution of the data. Categorical variables were assessed using the Chi-squared test. To evaluate the association between continuous cytokine levels and virologic suppression status, binary logistic regression was performed. Age and gender were controlled for through matching at the study design stage, thereby minimizing potential confounding. No additional covariates were adjusted for in the regression model. All analyses were two-tailed, and a p-value <0.05 was considered statistically significant. No subgroup analyses or interaction terms were applied; age and gender matching minimized baseline variability. Analyses focused on cytokine differences between predefined virologic groups. All participants had complete cytokine and viral load data; thus, no imputation or adjustments for missing data were necessary. Analysis reflected purposive age- and gender-matched sampling to minimize confounding and ensure group comparability. No formal sensitivity analyses were conducted.

**Ethical consideration:** ethical considerations were prioritized to ensure participant confidentiality and data security. IREC approval (Ref. No. 0003744) and permission from the CEO of MTRH (Ref: ELD/MTRH/R&P/10/2/V.2/2012) were obtained. Only the clinical officer identified participants from the HIV-1 adult clinic records; numeric codes were used to protect personal identifiers. Informed consent was obtained from all participants. Computer data was secured with a password, and hard copies were stored in lockable cabinets accessible only to the investigator.

## Results

**Study participants:** during January 2021-December 2022, 4,000 HIV-1-infected individuals on first-line cART at MTRH´s AMPATH module 1 clinic were screened. After excluding 3,812 based on viral load criteria, age mismatch, or incomplete records, 88 were eligible. Six declined participation, yielding a final cohort of 82 age- and gender-matched participants: 41 with persistent low-level viremia (50-500 copies/mL) and 41 virally suppressed (<50 copies/mL), all of whom were included in the final analysis.

**Demographic distribution:** among the 82 participants enrolled, an equal number of males (n=41; 50%) and females (n=41; 50%) were included. Each gender group had balanced representation across the two viral load categories, with 21 (26%) individuals exhibiting persistent low-level viremia (pLLV) and 20 (24%) being virally suppressed. Age stratification revealed a uniform distribution across the four predefined age groups (18-27, 28-37, 38-47, and 48-57 years). The highest representation was observed in the 38-47-year bracket for both males and females (15 participants each; 16.0%). No statistically significant association was observed between age group and viral load status within either gender. For males, the chi-square test yielded χ^2^= 0.468, p= 0.926, and for females, χ^2^= 1.700, p= 0.637-confirming adequate age matching and no confounding effect of age on viral suppression status.

**Figure 1:**
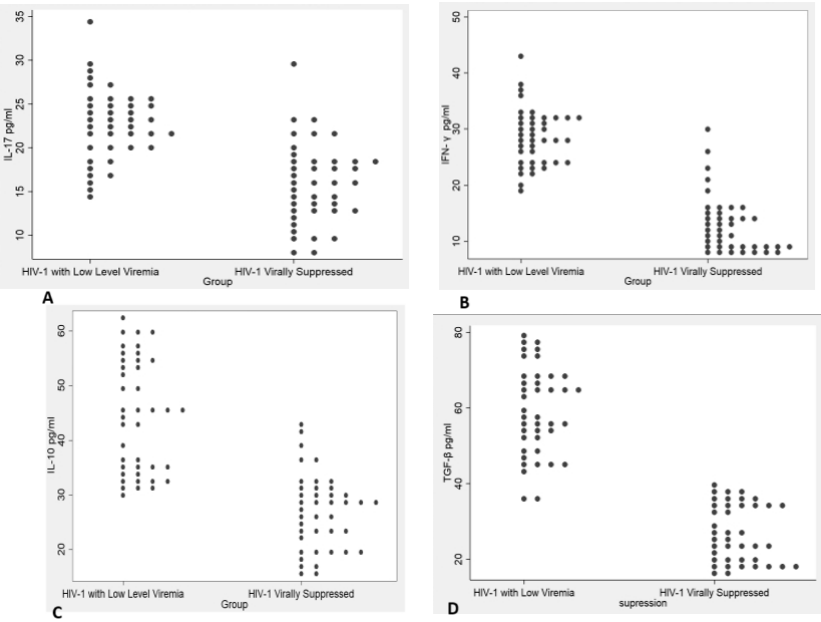
cytokines in persistent low-level viremia and virally suppressed participants on first-line combination antiretroviral therapy: A) IL-17; B) IFN-γ; C). IL-10; D) TGF-β

**Levels of IL-17, IFN-γ, IL-10, and TGF-β in HIV-1 participants on first-line cART with pLLV and HIV-1 suppressed participants:** the median (IQR) levels of IL-17, IFN-γ, IL-10, and TGF-β (pg/ml) in HIV-1 participants on first-line cART with pLLV and HIV-1 virally suppressed participants were 23.8 (21.3-25.8) vs 15.5 (12.7-18.2) p<.001; 28.3 (23.5-31.2) vs.11.4 (8.8-14.8), p<.001; 45.2 (34-53.7) vs 28.4 (22.6-31.6) p<.001 and 56.9 (50.0-67.8) vs 27.7 (19.5-34.7) respectively. Figure 1 shows IL-17, IFN-γ, IL-10, and TGF-β in pLLV and virally suppressed participants on first-line cART. All four cytokines, IL-17, IFN-γ, IL-10, and TGF-β, were significantly different between the two groups (p<0.001). Multiple logistic regression analysis was conducted to quantify the odds of being suppressed based on the effect of IL-17, IFN-γ, IL-10, and TGF-β in pg/ml. Table 1 shows the logistic regression of cytokine levels on viral suppression in HIV-1 participants on first-line cART. For each unit increase in TGF-β, the odds of being HIV-1 suppressed decreased by 66% (OR: 0.329; 95% CI: 0.035-3.061). Similarly, for each unit increase in IFN-γ, the odds decreased by 36% (OR: 0.636; 95% CI: 0.270-1.501), and for each unit increase in IL-17, the odds decreased by 6% (OR: 0.938; 95% CI: 0.691-1.273). Conversely, a unit increase in IL-10 increased the odds of suppression by 11% (OR: 1.106; 95% CI: 0.675-1.811). However, none of these were statistically significant (p>0.05). To address potential confounding, a multiple logistic regression was performed. The model, with a Cox and Snell R^2^ of 0.73, indicated that IL-17, IFN-γ, IL-10, and TGF-β levels explained 73% of the variability in viremia status, with 27% attributable to unexplored factors.

**Table 1:** logistic regression of cytokine levels on viral suppression in HIV-1 participants on first-line cART

Variable	Regression coefficient (β)	OR (95% CI)
IL-17	-0.064	0.938 (0.691-1.273)
IFN-γ	-0.452	0.636 (0.270-1.501)
IL-10	0.101	1.106 (0.675-1.811)
TGF-β	-1.112	0.329 (0.035-3.061)

cART: combination antiretroviral therapy; TGF-β: transforming growth factor beta

## Discussion

In our study, we found high IL-17 levels in HIV-1 participants on first-line cART with persistent low-level viremia (pLLV), consistent with findings by Mousavi *et al*. who also reported higher IL-17 levels in treated HIV-1 participants [15]. This supports the pro-inflammatory role of IL-17, which has been well-documented in some studies [16]. Elevated IL-17 suggests ongoing inflammation in pLLV participants, potentially due to persistent immune activation despite sub-optimal viral suppression, driven by continuous viral replication in reservoirs. Our findings differ from a recent study in Cameroon, which reported decreased IL-17 levels (13.2 pg/ml) in treated HIV-1 adolescents [17]. This reduction was interpreted as a sign of successful cART, indicating reduced inflammation in those with viral suppression. The discrepancy may be due to differences in study populations, such as age, which could influence immune responses to cART, as well as variations in treatment regimens, viral suppression, and treatment duration. Our study highlights that inflammation and viral proliferation may persist in cART-treated participants with pLLV, pointing to a form of immune dysregulation that persists despite treatment. Elevated IL-17 in these participants may reflect sustained inflammation, contributing to immune system dysfunction and potential disease progression, even with ongoing cART [16]. In our study, we observed elevated levels of IFN-γ in HIV-1 participants on first-line cART with persistent low-level viremia (pLLV), consistent with previous research [15]. Elevated IFN-γ is considered indicative of an active antiviral immune response, suggesting the immune system´s attempt to control persistent HIV-1 replication despite cART. This aligns with the idea that IFN-γ is part of the host's defense mechanism, even in the presence of low-level viremia. Contrarily, a study by Ka´e *et al*. in Cameroon [17], showed an inverse correlation between IFN-γ levels and viral load, with lower IFN-γ associated with effective cART-induced immune reconstitution. The discrepancy may arise from differences in study populations, stages of HIV-1 infection, cART duration, or levels of viral suppression.

The elevated IFN-γ levels in our study suggest a complex interaction between the immune response and HIV-1 replication. IFN-γ plays a key role in antiviral immunity, but persistent high levels can lead to immune dysfunction, including CD4+ T cell depletion, a hallmark of HIV-1 progression [18,19]. In participants with pLLV, elevated IFN-γ may indicate an immune system under strain, attempting to control the virus despite incomplete suppression, potentially contributing to immune system damage and immunosuppression. Our findings align with Kang *et al*. [20], who suggested that elevated IFN-γ could reflect immunosuppression in HIV-1 participants, particularly those with pLLV. Persistent immune activation without effective viral control may lead to immune exhaustion and dysfunction, underscoring the need for improved management strategies to achieve complete viral suppression and prevent immune dysregulation. These results highlight the complex role of IFN-γ in HIV-1 pathogenesis, where its elevation in pLLV may signal underlying immunosuppression despite its crucial role in antiviral immunity. Elevated IL-10 levels were observed in HIV-1 participants on first-line cART, a finding consistent with previous research. For example, Twizerimana *et al*. in Rwanda reported that IL-10 levels correlated with viral load, noting a decrease in IL-10 from 120 to 48 pg/ml after 6 months of cART, with higher IL-10 levels linked to increased viral loads [21]. This suggests that IL-10, an anti-inflammatory cytokine, may be upregulated in response to ongoing viral replication, reflecting the immune system's attempt to modulate inflammation amidst persistent infection. However, our findings diverge from those of Osuji *et al*. in Nigeria, who reported reduced IL-10 levels following cART initiation [22]. They suggested that lower IL-10 levels post-cART indicate immune recovery, as a decrease in IL-10 is associated with the restoration of normal immune function after effective viral suppression. This discrepancy may stem from differences in the stages of immune recovery and viral suppression across patient populations. In participants with better viral suppression, a reduction in IL-10 may signal immune homeostasis, whereas in our cohort, elevated IL-10 levels could reflect ongoing immune dysregulation due to persistent low-level viremia despite treatment. Additionally, research by Harper *et al*. in the USA highlighted IL-10´s role in immune modulation, especially in promoting regulatory T cell (Treg) development, which helps control immune responses and prevent excessive inflammation [23]. While IL-10 is crucial for maintaining immune tolerance, excessive levels can lead to immune suppression.

Studies by Lobo-Silva *et al*. and Kahle *et al*. also indicated that elevated IL-10 levels can contribute to immune suppression, hindering the body's ability to effectively control viral replication [24,25]. Consistent with this Musa *et al*. in Kenya observed a correlation between higher IL-10 levels and increased viral load and disease progression [26]. Thus, elevated IL-10 levels in our study may indicate a compromised immune response in HIV-1 participants with persistent low-level viremia, where immune suppression limits the body's ability to mount a robust defense against ongoing viral replication. This suggests that while IL-10 may modulate the immune response, its excess may contribute to immune suppression, impairing immune reconstitution and facilitating disease progression. Elevated TGF-β levels were observed in HIV-1 participants on first-line cART with persistent low-level viremia (pLLV), contrasting with previous studies that reported reduced TGF-β levels following cART initiation. For instance, Musa *et al*. noted a decrease in TGF-β from 77.7 to 64.3 pg/ml over 12 months [26], and Osuji *et al*. found a similar reduction in TGF-β levels with cART, suggesting treatment benefits in reducing immune dysfunction and controlling inflammation [22]. However, our findings indicate that, despite cART, participants with pLLV do not experience the same degree of immune reconstitution or viral suppression.

The discrepancy between our results and those of Prudhula *et al*. who reported lower TGF-β levels (26.67 pg/ml) post-cART [27], highlights the variability in the impact of cART on TGF-β across different populations. Factors such as patient demographics, treatment regimens, viral load suppression, and treatment duration may explain these differences. While cART may lower TGF-β in many participants, those with persistent low-level viremia may still exhibit elevated levels, indicating incomplete immune reconstitution or persistent immune activation. Transforming growth factor beta (TGF-β) plays a crucial role in immune modulation by regulating inflammatory responses and maintaining immune tolerance [28]. However, it also contributes to viral reservoir persistence, complicating efforts to fully suppress HIV-1 replication. In participants with pLLV, TGF-β may sustain viral reservoirs, enabling ongoing viral replication despite apparent viral suppression. Furthermore, TGF-β induces regulatory T cells (Tregs), which, while important for controlling immune responses, may promote immune evasion and suppression. Jiang *et al*. 2018 suggested that TGF-β-induced Tregs could exacerbate HIV-1 progression by limiting the immune system's ability to combat the virus, contributing to chronic immune dysfunction [29]. Thus, elevated TGF-β levels likely reflect ongoing immune dysregulation in participants with pLLV despite cART. While cART may reduce TGF-β in some participants, its effects may be insufficient in those with pLLV, potentially contributing to viral reservoir persistence and chronic immune activation. These findings underscore the complexity of the immune response in such participants, where elevated pro-inflammatory cytokines indicate persistent immune activation despite the effectiveness of cART in reducing viral load.

**Limitations of the study:** study limitations include potential confounding from unverified self-reported comorbidities and underlying immune disorders, which may have influenced cytokine levels. Additionally, the duration since cART initiation was not considered, possibly affecting the variability of the findings.

**Cautious interpretation of results:** this study examined the association between persistent low-level viremia (pLLV) and cytokine profiles in HIV-1 individuals on cART. Findings revealed significantly elevated levels of both pro-inflammatory (IL-17, IFN-γ) and anti-inflammatory (IL-10, TGF-β) cytokines in pLLV participants compared to those who were virally suppressed. These results should be interpreted in light of the study´s aims, limitations, multiple statistical comparisons, and existing literature.

**Generalizability (external validity) of the study results:** while the study offers important insights into cytokine profiles among pLLV patients on first-line cART at MTRH, its generalisability is limited. The findings are most applicable to similar resource-limited settings in sub-Saharan Africa. Extrapolation to other contexts with differing healthcare systems, treatment protocols, or patient demographics should be made cautiously. Multi-centre studies with larger, diverse cohorts are needed to validate these results and clarify the utility of cytokine profiling in HIV-1 virologic monitoring.

**Recommendation:** cytokines in HIV-1 participants could offer valuable insights into viral suppression failure. Prospective studies of other pro/anti-inflammatory cytokines' relation with viremia are recommended in HIV-1 participants on cART.

## Conclusion

Persistent low-level viremia (pLLV) in HIV-1 participants is related to levels of both pro-inflammatory (IL-17, IFN-γ) and anti-inflammatory (IL-10, TGF-β) cytokines.

### What is known about this topic

- Cytokine Imbalance in HIV-1 participants on combination antiretroviral therapy: HIV-1 infection causes cytokine imbalance, increasing pro-inflammatory cytokines, and despite combination antiretroviral therapy, immune activation and inflammation persist, contributing to low-level viremia;
- Persistent low-level viremia and immune response: low-level viremia in combination with antiretroviral therapy-treated participants is linked to chronic inflammation, sustaining immune activation and altering cytokine profiles, which may lead to complications like cardiovascular and neurocognitive disorders.

### What this study adds

- This study enhances understanding of cytokine profiles in HIV-1 participants on combination antiretroviral therapy with persistent low-level viremia at a tertiary hospital in western Kenya;
- It provides insights into the link between chronic inflammation, cytokine imbalances, and low-level viremia, potentially identifying region-specific immune activation patterns and biomarkers for improved clinical management.
